# Electroporation of human microvascular endothelial cells: evidence for an anti-vascular mechanism of electrochemotherapy

**DOI:** 10.1054/bjoc.2000.1625

**Published:** 2001-02

**Authors:** M Cemazar, C S Parkins, A L Holder, D J Chaplin, G M Tozer, G Sersa

**Affiliations:** Department of Tumor Biology, Institute of Oncology, Zaloska 2, SI-1000, Ljubljana, Slovenia; Tumour Microcirculation Group, Gray Laboratory Cancer Research Trust, Mount Vernon Hospital, PO Box 100, Northwood, Middlesex, HA6 2JR, UK

**Keywords:** endothelial cells, anti-vascular action, electrochemotherapy, electroporation, bleomycin, cisplatin

## Abstract

Recent studies have indicated that the antitumour effectiveness of electrochemotherapy, a combination of chemotherapeutic drugs with application of high voltage electric pulses applied to the tumour nodule (electroporation), result in a significant reduction in tumour blood flow and may therefore be mediated by an anti-vascular mechanism. The aim of this study was to evaluate the cytotoxicity of electroporation with bleomycin or cisplatin on cultured human microvascular endothelial cells (HMEC-1). The sensitivity of HMEC-1 cells to a 5 min treatment by electroporation with bleomycin or cisplatin (8 electric pulses, pulse duration 100 μs, frequency 1 Hz, electric field intensity 1400 V cm^–1^) was compared to the sensitivity of cells treated continuously for 3 days with drugs alone. HMEC-1 cells were moderately sensitive to continuous exposure to cisplatin, but showed greater sensitivity to bleomycin. Combination of a 5 min drug exposure with electric pulses increased cytotoxicity of cisplatin by ∼10-fold for cisplatin and ∼5000-fold for bleomycin. The electroporation of HMEC-1 cells with bleomycin for a 5 min exposure was ∼250-fold better than a continuous exposure to the drug alone. The results of this study indicate that the anti-tumour action of electrochemotherapy is likely to be due, in part, to the highly sensitive response of vascular endothelial cells. Further studies are necessary to identify the determinants of endothelial response and its relationship to the anti-vascular action of electrochemotherapy in vivo. © 2001 Cancer Research Campaign http://www.bjcancer.com


*British Journal of Cancer* (2001) **84**(4), 565–570
© 2001 Cancer Research Campaign
doi: 10.1054/ bjoc.2000.1625, available online at http://www.idealibrary.com on IDEAL

### Electroporation of human microvascular endothelial cells: evidence for an anti-vascular mechanism of electrochemotherapy

**M Cemazar[1,2], CS Parkins[2], AL Holder[2], DJ Chaplin[2], GM Tozer[2] and G Sersa[1]**

1Institute of Oncology, Department of Tumor Biology, Zaloska 2, SI-1000 Ljubljana, Slovenia; 2Tumour Microcirculation Group, Gray Laboratory Cancer Research Trust, Mount Vernon Hospital, PO Box 100, Northwood, HA6 2JR, Middlesex, UK

**Summary** Recent studies have indicated that the antitumour effectiveness of electrochemotherapy, a combination of chemotherapeutic drugs with application of high voltage electric pulses applied to the tumour nodule (electroporation), result in a significant reduction in tumour blood flow and may therefore be mediated by an anti-vascular mechanism. The aim of this study was to evaluate the cytotoxicity of electroporation with bleomycin or cisplatin on cultured human microvascular endothelial cells (HMEC-1). The sensitivity of HMEC-1 cells to a 5 min treatment by electroporation with bleomycin or cisplatin (8 electric pulses, pulse duration 100 μs, frequency 1 Hz, electric field intensity 1400 V $cm^{-1}$) was compared to the sensitivity of cells treated continuously for 3 days with drugs alone. HMEC-1 cells were moderately sensitive to continuous exposure to cisplatin, but showed greater sensitivity to bleomycin. Combination of a 5 min drug exposure with electric pulses increased cytotoxicity of cisplatin by ~10-fold for cisplatin and ~5000-fold for bleomycin. The electroporation of HMEC-1 cells with bleomycin for a 5 min exposure was ~250-fold better than a continuous exposure to the drug alone. The results of this study indicate that the anti-tumour action of electrochemotherapy is likely to be due, in part, to the highly sensitive response of vascular endothelial cells. Further studies are necessary to identify the determinants of endothelial response and its relationship to the anti-vascular action of electrochemotherapy in vivo.
© 2001 Cancer Research Campaign htt://www.bjcancer.com

**Keywords**: endothelial cells; anti-vascular action; electrochemotherapy; electroporation; bleomycin; cisplatin

Electrochemotherapy is a combined modality treatment using chemotherapy and electroporation (Mir and Orlowski, 2000). Electroporation is a physical method, performed by application of high voltage direct current electric pulses to cells in vitro or tissues in vivo, to increase cell uptake of molecules such as DNA, antibodies, enzymes, dyes and drugs by permeabilization of the plasma membrane (Mir and Orlowski, 2000; Sersa, 2000). In electrochemotherapy, the optimal antitumour effectiveness is achieved when treatment is given at the time of the highest extracellular concentration of hydrophilic chemotherapy drugs, thereby increasing transport through plasma membrane towards the intracellular targets for cytotoxicity (Mir and Orlowski, 2000). The chemotherapeutic drugs, bleomycin and cisplatin, have proven to be effective in electrochemotherapy of experimental tumour cells, both in vitro and in vivo (Mir and Orlowski, 2000; Sersa, 2000) and also in electrochemotherapy of accessible tumour nodules of various malignancies in cancer patients (Heller et al, 2000). Neither drugs alone nor high voltage electric pulses as single treatment affected tumour growth (Heller et al, 2000; Mir and Orlowski, 2000; Sersa, 2000).

Antitumour effectiveness of electrochemotherapy is primarily due to the increased uptake of the chemotherapeutic drugs into the tumour cells, caused by electroporation. The increased accumulation has been confirmed for platinum (component of cisplatin) and radioactively labelled bleomycin into tumour cells in vivo after treatment with electrochemotherapy (Belehradek et al, 1994; Cemazar et al, 1999). However, several other mechanisms have been demonstrated to be involved in tumour response to electrochemotherapy, e.g. involvement of immune system and changes in tumour blood flow (Mir et al, 1995; Sersa et al, 1997a,b). The vascular targeting action of electrochemotherapy was only speculated. The immune status of experimental mice was shown to be positively correlated with the incidence of complete responses of tumours treated with electrochemotherapy (Sersa et al, 1997a) and potentiation of tumour response to electrochemotherapy was achieved by adjuvant immunotherapy, either with IL-2 or TNF-α (Mir et al, 1995; Sersa et al, 1997b). We have previously shown that the application of electric pulses alone result in substantial, but transient, reduction of tumour perfusion (Sersa et al, 1999a) and furthermore that electrochemotherapy with bleomycin causes rapid cessation of tumour perfusion (Sersa et al, 1999b). In addition, electrochemotherapy prolonged entrapment of cisplatin for up to 8 hours within the tumour (Cemazar et al, 1999). We hypothesize that the anti-tumour action of electrochemotherapy may be partly due to its antivascular action. It is our hypothesis that damage to the endothelial cells, which are electroporated during application of electric pulses, leads to the reduction of tumour perfusion, which contributes to the antitumour effectiveness of electrochemotherapy.

The aim of the present study was to investigate the role of endothelial cells in the antivascular action of electrochemotherapy. The sensitivity of human microvascular endothelial cells, HMEC-1, to electroporation with cisplatin or bleomycin was determined

*Received 20 March 2000*
*Revised 20 November 2000*
*Accepted 21 November 2000*

*Correspondence to:* M Cemazar in vitro and compared to sensitivity of the cells to the treatment with the drugs alone.

### MATERIALS AND METHODS

### Chemicals

Cisplatin (Platamine, Pharmacia & Upjohn, Milan, Italy) was dissolved in sterile $H_2O$ to obtain a concentration of 6.7 mM. The final concentrations were prepared in Dulbecco's modified Eagles medium (D-MEM; Gibco Life Technologies, Paisley, UK). Bleomycin (Bleo-Kyowa, Kyowa Hakko, Slough, UK) was dissolved in D-MEM at a concentration of 10 mM. All further dilutions were also prepared in D-MEM. Propidium iodide (Sigma-Aldrich Company Ltd, Poole, UK) was prepared in phosphate-buffered saline in a concentration of 0.1 mM.

### Cells

Human dermal microvascular endothelial cells (HMEC-1) were generously provided by Dr FJ Candal (Center for Disease Control, Atlanta, USA). This cell line was immortalized by transfection with a plasmid containing simian virus 40A gene product, large T antigen (Ades et al, 1992). HMEC-1 cells were grown as monolayer in D-MEM supplemented with 10% fetal calf serum (FCS, Sigma) in a humidified incubator at atmospheric oxygen supplemented with 5% $CO_2$ at 37˚C. They were routinely subcultured twice per week. When grown in monolayer the cells exhibited typical cobblestone morphology.

### Determination of electro-permeabilization and electro-sensitivity

Electro-permeabilization of plasma membrane was measured by cellular uptake of propidium iodide. Electro-sensitivity (survival of cells treated with electric pulses) was determined by 3-(4,5-dimethylthiazol-2-yl)-2,5-diphenyl-tetrazolium bromide (MTT) assay. The cells were prepared from exponential growth phase, trypsinized and washed twice, first in the D-MEM supplemented with 10% FCS for inactivation of trypsin (Sigma), and then in the serum-free D-MEM. Cell suspensions ($2.2 \times 10^7$ cells $ml^{-1}$ in 90 µl) were prepared containing 10 µl propidium iodide (100 µM) for permeability assay or with D-MEM for MTT cytotoxicity assay. The cells were placed between the two parallel stainless-steel electrodes (length 6 mm, width 6 mm, gap 2 mm) connected to the electroporator (Jouan GHT 1287, France) and subjected to 8 square wave electric pulses (pulse width 100 µs, repetition frequency 1 Hz) of different electric field intensities, ranging from 400 to 1800 V $cm^{-1}$ (Figure 1). After exposure to electric pulses, the cells were incubated for 5 min at room temperature (24˚C). This incubation time was shown to be the optimal, since it allows resealing of plasma membrane and it does not affect cell viability due to the evaporation of medium and lack of nutrients (Rols and Teissie, 1998). To measure the propidium iodide uptake, 25 µl of cell suspension was resuspended in 1 ml of 0.01 M phosphate buffered saline (pH 7.4) and analysed immediately by FACSort (Becton Dickinson, Mountain View, CA, USA). The percentage of stained cells was determined in comparison to the control cells that were not subjected to electric pulses. The electro-sensitivity of cells was determined by MTT assay performed 3 days after plating in 96-well microtitre plates. Briefly, at the end of incubation time MTT solution (25 µl of 5 mg $ml^{-1}$ solution) was added to each well and the microtitre plates were further incubated for 3 h at 37˚C. The formed formazan crystals were dissolved in 100 µl of dimethyl sulphoxide (Sigma). The microtitre plates were shaken for 99 s to ensure adequate solubilization and the absorbance of the resulting solution was measured at 570 nm using an Anthos microplate reader (Anthos Labtec, Salzburg, Austria). The survival of cells treated with electric pulses was presented as a percentage of the absorbency obtained from control untreated cells.

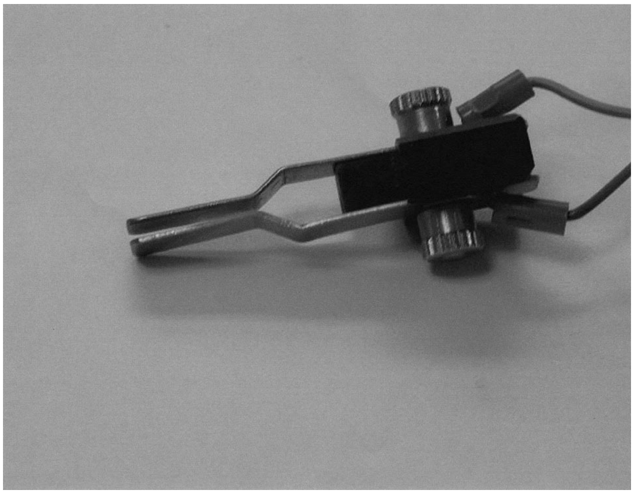

**Figure 1** Photograph of the electrodes used in the experiments. The gap between the electrodes was 2 mm and 50 µl of cell suspension was put between the electrodes using an automatic pipette

### Cytotoxicity assay for continuous exposure of cells to drugs and combination of electroporation and drugs

To determine the sensitivity of HMEC-1 cells to continuous (3 days) exposure to cisplatin or bleomycin, the cells were plated in 96-well microtitre plates and incubated with cisplatin (1.67 to 16.7 µM) or bleomycin (0.1 nM to 10 µM). The sensitivity of the cells to combined treatments of cisplatin or bleomycin with electric pulses (electroporation) was determined as described above for electro-sensitivity, except that the cells were mixed with cisplatin or bleomycin solution instead of the serum-free D-MEM at the time of electroporation. One half of the cell suspension was exposed to electric pulses (1400 V $cm^{-1}$) and the other half served as a control for cisplatin or bleomycin treatment alone. The cisplatin concentrations used were ranging from 16.7 to 670 µM and the bleomycin concentrations from 0.1 nM to 100 µM. The cells were exposed to the drugs for 5 min, since this incubation time was shown to be the optimal, since it allows resealing of plasma membrane and it does not affect cell viability due to the evaporation of medium and lack of nutrients (Rols and Teissie, 1998). After that, cells suspension was diluted with D-MEM supplemented with 10% FCS and plated in 96-well microtitre plates. The survival of cells treated with electroporation after exposure to drugs was normalized to electric pulses treatment alone. The $IC_{50}$ value (the concentration of cisplatin or bleomycin that causes 50% reduction in cell survival) was determined for each treatment group.

 © 2001 Cancer Research Campaign

### Statistical analysis

Data were tested for normality using Kolmogorov-Smirnov test. Data were normally distributed and are therefore represented as an arithmetic mean ± standard error of the mean. Statistical analysis was done using SigmaStat statistical software (SPSS Inc).

### RESULTS

### Electro-permeabilization and electro-sensitivity

In order to determine the optimal electric field intensity, without significant cytotoxicity, both electro-permeabilization (propidium iodide) and electro-sensitivity (MTT assay) of HMEC-1 cells were measured over a range of electric field intensities, which is the critical parameter for obtaining electro-permeabilization (Figure 2). Electro-permeabilization of HMEC-1 cells, at an intensity of 1000 V cm$^{-1}$, was found to induce 30% of cells to be permeabilized. At the highest intensity of 1800 V cm$^{-1}$ only 85% of cells were permeabilized. This indicates a relatively high cellular resistance to electroporation although the survival of cells was reduced to 57% at this intensity. Therefore, 1400 V cm$^{-1}$ was selected for the subsequent electrochemotherapy experiments. At this electric field intensity, only 60% of cells were permeabilized, but the survival of cells was still approximately 80%.

### Cell survival after continuous exposure of cells to drugs and combination of electroporation and drugs

To determine the sensitivity of HMEC-1 cells to cisplatin alone, cells were continuously exposed to the drug and cell survival determined after 3 days. Continuous exposure of HMEC-1 cells to cisplatin resulted in significantly reduced cell survival at doses higher than 0.67 μM. The $IC_{50}$ value for cells to cisplatin was 1.8 μM. In experiments using the combination of electroporation and drugs, the cells were exposed to cisplatin for only 5 min. The $IC_{50}$ value for cells treated with cisplatin alone was 180 μM (Figure 3). Exposure of cells to electric pulses increased cisplatin cytotoxicity 10-fold ($IC_{50}$ value reduced to 18 μM) compared to the $IC_{50}$ value of cells exposed to the cisplatin for 5 min. In comparison to cell survival after continuous exposure to cisplatin, the $IC_{50}$ value for the combination of electroporation and drugs (5 min) treated cells was ~5-times higher.

The survival of HMEC-1 cells after continuous (3 days) exposure to bleomycin was reduced at the doses higher than 10 nM. The $IC_{50}$ value of HMEC-1 cells continuously exposed to bleomycin was 1 μM. The $IC_{50}$ value cells treated with bleomycin for 5 min was 20 μM (Figure 4). The potentiation of bleomycin cytotoxicity, when cells were exposed to electric pulses was very high, ~5000-fold, when compared to the $IC_{50}$ value of cells exposed to the bleomycin for 5 min. The $IC_{50}$ value of cells treated with combination of electroporation and bleomycin was 4 nM, which was ~700-fold lower than compared to cells that were continuously exposed to bleomycin.

### DISCUSSION

The results of this study demonstrate that electroporation with cisplatin or bleomycin significantly enhances the cytotoxicity to

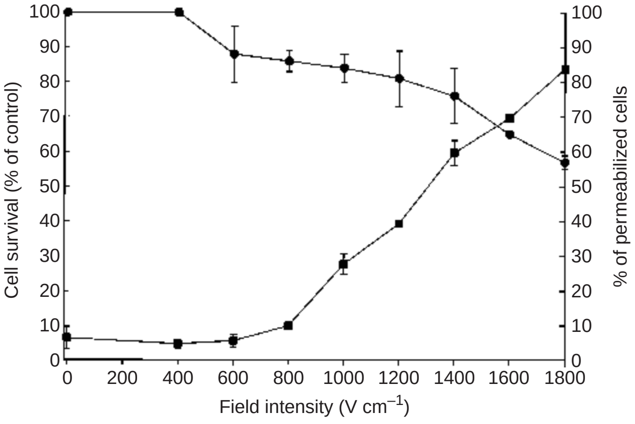

**Figure 2** Incidence of electro-permeabilization (■) and electro-sensitivity (●) in HMEC-1 cells by increasing electroporation field strength. Electro-permeabilization was measured by propidium iodide uptake, electro-sensitivity by MTT assay. Data are mean ±1 standard error of the mean pooled from 3 independent experiments

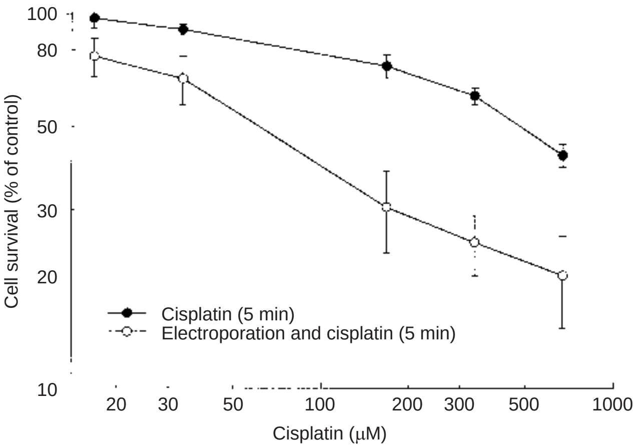

**Figure 3** Cell survival after 5 min. (●) exposure of HMEC-1 cells to cisplatin. In electrochemotherapy protocol (1400 V cm$^{-1}$), cells were exposed to the drug for 5 minutes (O). Data are mean ±1 standard error of the mean pooled from 3 independent experiments

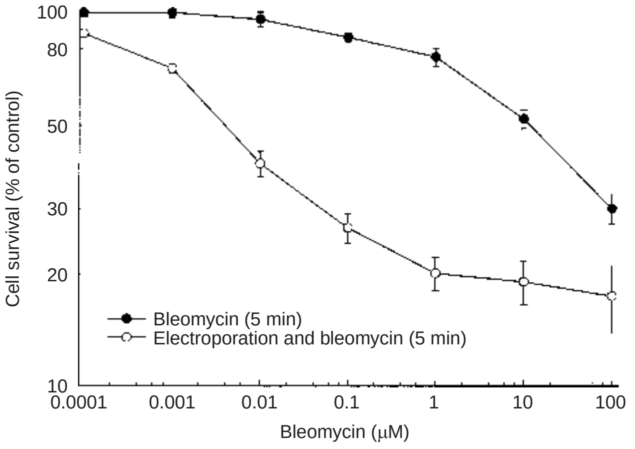

**Figure 4** Cell survival after 5 min. (●) exposure of HMEC-1 cells to bleomycin. In electrochemotherapy protocol (1400 V cm$^{-1}$), cells were exposed to the drug for 5 minutes (O). Data are mean ±1 standard error of the mean pooled from 3 independent experiments human endothelial HMEC-1 cells, even after very short drug exposure of cells. It is therefore probable that in vivo electrochemotherapy, especially with bleomycin, may directly damage the vascular endothelium and account for its antivascular actions, such as reduced tumour blood flow and infiltration by host blood cells (Sersa et al, 1999a, b). The antivascular action of electrochemotherapy would be in addition to other, non-vascular, mechanisms previously reported (Sersa et al, 1997a, b; Cemazar et al, 1999).

Continuous exposure of human endothelial cells to bleomycin or cisplatin reduced cell survival substantially in a dose-dependent manner although this effect was more pronounced with bleomycin than with cisplatin. In comparison to the $IC_{50}$ values for several human and murine tumour cell lines, treated with either of these drugs (Tables 1 and 2), the values for HMEC-1 cells appear relatively resistant, especially to cisplatin (Lazo et al, 1989; Hait et al, 1994; Kambe et al, 1996; Dirix et al, 1997; Cemazar et al, 1998a,b). This is consistent with a report of relatively little antivascular action of cisplatin in vivo (Clements et al, 1999). In contrast, the present result for bleomycin indicates that HMEC-1 cells show an equal, or enhanced, sensitivity to that reported for tumour cells with the maximum value of ~ 200-fold more sensitive than tumour cell lines (Table 2) (Hait et al, 1994; Dirix et al, 1997; Cemazar et al, 1998a). At present, only Dirix et al have reported on the sensitivity of two different endothelial cell lines to bleomycin (Dirix et al, 1997). In that study, the response of human umbilical vein endothelial cells (HUVEC) were compared to HMEC and demonstrated a dose-response in survival with bleomycin dose. However, the effect was marginal and the $IC_{50}$ for both HUVEC and HMEC cells were in the same range shown by the most resistant tumour cell lines (Table 2). These observations are in contrast to the present results and probably due to the difference in endothelial cell lines and also to the reduced proliferation status (semi-confluent and confluent) of the cultures used to assess bleomycin cytotoxicity (Dirix et al, 1997) in comparison to the exponential growth phase used in the present study.

In the present study, electric pulses were used as a drug delivery system for bleomycin and cisplatin, which both are reported to have restricted transport through plasma membrane (Belehradek et al, 1994; Cemazar et al, 1999). Increased drug uptake and potentiation of cytotoxicity has been demonstrated in vitro for several tumour cell lines treated with cisplatin, bleomycin, peplomycin and others, following exposure to electric pulses (Orlowski et al, 1988; Sersa et al, 1995; Kambe et al, 1996; Cemazar et al, 1998a; Kuriyama et al, 1999). When used in combination with application of electric pulses the drug dosage can be greatly reduced for equivalent or higher cytotoxic effect, compared to drug treatment alone (Orlowski et al, 1988; Sersa et al, 1995; Kambe et al, 1996; Cemazar et al, 1998a; Kuriyama et al, 1999). When electrochemotherapy is performed in vivo, the tumour perfusion and intravascular concentration of the drug are additional factors to be considered in achieving optimal tumour cytotoxicity. However, electroporation of solid tumours will also involve treatment of non-tumour cells within the treatment field, including the endothelial cells lining the tumour vasculature. Our in vitro results demonstrate that endothelial cells are electroporated at the same field intensity previously shown to be effective for tumour cytotoxicity in vivo (Sersa et al, 1995; Miklavcic et al, 1998). However, compared to the tumour cells in vitro they appeared to be slightly more resistant to electric pulses (Cemazar et al, 1998c). The reasons for this are currently unclear. To our knowledge, the present study is the first report demonstrating the high sensitivity of human endothelial cells to electroporation with bleomycin and to lesser extent to electroporation with cisplatin compared to the tumour cell lines used in the electrochemotherapy protocol (Tables 1, 2). Although the direct comparison of cell sensitivity to the combination of electroporation and bleomycin or cisplatin would require that the experiments would be done in the same study, according to those studies there is some evidence for endothelial cells being more sensitive to bleomycin than tumour cells. When electroporation was combined with bleomycin, cell cytotoxicity was assessed using MTT assay, while clonogenic assay was used in most of the studies testing combination of electroporation and cisplatin (Tables 1, 2) (Sersa et al, 1995; Kambe et al, 1996; Cemazar et al, 1998a).

The present in vitro data for endothelial cell cytotoxicity are consistent with our previous demonstration that tumour blood flow is completely shut down 12 hours after electrochemotherapy with bleomycin (Sersa et al, 1999b). The severity of the overall antivascular action of electrochemotherapy is likely to be due to a combination of actions mediated at different stages of the cytotoxic mechanism. For example, the increased uptake of drugs, following electroporation of plasma membrane, has been demonstrated in tumour cells, by increased uptake of radioactively labelled bleomycin and by increased DNA platination (Belehradek et al, 1994; Cemazar et al, 1999). It is unlikely to be cell-specific in vivo and may account, in part, for the antivascular action of electrochemotherapy. Secondly, the infiltration of host immune cells

**Table 1** $IC_{50}$ values of tumour and endothelial cell lines treated with cisplatin (continuous exposure) or combination of electroporation and cisplatin

| Cell line type | Cell line (assay[a]) | $IC_{50}$(μM) cisplatin | $IC_{50}$ (μM) combination | Reference |
|---|---|---|---|---|
| Tumour | EAT (MTT) | 0.4 | ND[b] | Cemazar (unpub.) |
| | SA-1 (clonogenic) | 0.9 | 20.0 | Cemazar (unpub.) |
| | TBL.C12 (clonogenic) | 0.18 | 7.0 | Cemazar (unpub.) |
| | TBL.C12 Pt (clonogenic) | 1.5 | 7.0 | Cemazar (unpub.) |
| | B16 (clonogenic) | 1.6 | 11.0 | Sersa et al, 1995 |
| | A253 (Coulter counter) | 0.13 | ND | Lazo et al, 1989 |
| | IGROV 1 (clonogenic) | 0.13 | 20.0 | Cemazar et al, 1998b |
| | IGROV 1/DDP (clonogenic) | 2.0 | 110.0 | Cemazar et al, 1998b |
| | LS174T (MTT) | 0.9 | 40.0 | Kambe et al, 1996 |
| Endothelial | HMEC-1 (MTT) | 12.0 | 40.0 | Present study |

[a]Assay used to assess cell cytotoxicity. [b] Not determined.

 *© 2001 Cancer Research Campaign*

**Table 2** $IC_{50}$ values of tumour and endothelial cell lines treated with bleomycin (continuous exposure) or combination of electroporation and bleomycin

| Cell line type | Cell line (assay[a]) | $IC_{50}$ (μM) bleomycin | $IC_{50}$ (μM) combination | Reference |
|---|---|---|---|---|
| Tumour | SA-1 (MTT) | 20.0 | 0.025 | Cemazar et al, 1998a |
| | EAT (MTT) | 200.0 | 0.7 | Cemazar et al, 1998a |
| | L1210 (clonogenic) | 0.9 | ND[b] | Hait et al, 1994 |
| | MCF (clonogenic) | 17.0 | ND | Hait et al, 1994 |
| | 9L (clonogenic) | 4.2 | ND | Hait et al, 1994 |
| | C6 (clonogenic) | 29.0 | ND | Hait et al, 1994 |
| | DC-3F (clonogenic) | ND | 0.0015 | Orlowski et al, 1988 |
| | Colon 26 (MTT) | ND | 0.01 | Kuriyama et al, 1999 |
| | MC38 (MTT) | ND | 0.06 | Kuriyama et al, 1999 |
| | Colo320 (MTT) | >70.0 | ND | Kambe et al, 1996 |
| Endothelial | HUVEC (Alamar Blue) | >86.0 | ND | Dirix et al, 1997 |
| | HMEC (Alamar Blue) | 76.0 | ND | Dirix et al, 1997 |
| | HMEC-1 (MTT) | 1.0 | 0.004 | Present study |

[a]Assay used to assess cell cytotoxicity. [b] Not determined.

into the tumour has been shown to be responsible for a degree of tumour cytotoxicity, as evidenced by the change in tumour response to electrochemotherapy applied in immunodeficient animals (Sersa et al, 1997a). Overall, the changes in endothelial function and survival are not unexpected, in relation to tumour cell response, and are consistent with the observed changes in tumour blood flow, as measured using $^{86}$RbCl extraction and Patent Blue technique (Sersa et al, 1999a,b).

The finding that electrochemotherapy has an antivascular action is important since it could identify other approaches to increase the effectiveness of electrochemotherapy treatment of tumours. Electrochemotherapy is being investigated in several trials on cancer patients. Clinical studies on accessible tumour nodules of different tumour types, such as malignant melanoma, basal cell carcinoma, squamous cell carcinoma, adenocarcinoma and others have demonstrated that electrochemotherapy with both, bleomycin or cisplatin is very effective, resulting in high incidence of complete responses (Heller et al, 2000). At present, a major limitation to this treatment is its applicable to small (< 10 mm diameter) cutaneous and subcutaneous nodules that can be placed between the electrodes. This limitation can be overcome by either repetitive application so that the whole tumour area is covered, or by repeating the therapy on any residual tumour mass or by the use of needle electrodes that enable better distribution of the electric field within each tumour (Gilbert et al, 1997; Heller et al, 2000).

It is now clear that improvements in outcome of electrochemotherapy could potentially be achieved by exploiting its antivascular action. Electrochemotherapy could be applied to blood vessels that supply the tumours, if accessible, and achieve cessation of blood supply similar to vascular embolization, a technique already in practice (Acunas and Rozanes, 1999). Intra-arterial injection of either bleomycin or cisplatin, into the vessel supplying the tumour, immediately before electroporation of the tumours would spare the systemic cytotoxicity of the drug because the drug dose used for electrochemotherapy could be reduced relative to the intravenous or intratumour dose necessary for the same tumour effect. These two approaches would exploit the advantages associated with anti-vascular strategies; namely, that the incidence of damaged endothelial cells would result in cessation of flow to an entire vessel segment, consequently enhancing cytotoxicity of tumour cells (Denekamp, 1990).

In conclusion, our demonstration that electroporation with cisplatin or bleomycin is highly cytotoxic for a human endothelial HMEC-1 cell provides evidence for a potential mechanism which explains the observed antivascular actions of electrochemotherapy. Future studies to identify the determinants of endothelial response, and their relationship to antivascular action, will result in increased cytotoxicity by electrochemotherapy.

### ACKNOWLEDGEMENT

This work was supported by the Ministry of Science and Technology of the Republic of Slovenia and UICC fellowship grant No 1031/99. GMT, CSP and ALH are supported by the Cancer Research Campaign of United Kingdom. Current address for DJC; Oxygene INC, One Cotley, Suite 602, Boston MA 02116.

### REFERENCES

Acunas B and Rozanes I (1999) Hepatocellular carcinoma treatment with transcatheter arterial chemoembolization. *Eur J Radiol* **32(1)**: 86–89

Ades EW, Candal FJ, Swerlick RA, George VG, Summers S, Bosse DC and Lawley TJ (1992) HMEC-1: establishment of an immortalized human microvascular endothelial cell line. *J Invest Dermatol* **99**: 683–690

Belehradek Jr J, Orlowski S, Ramirez LH, Pron G, Poddevin B and Mir LM (1994) Electropermeabilization of cells in tissues assessed by the qualitative and quantitative electroloading of bleomycin. *Biochim Biophys Acta* **1190**: 155–163

Cemazar M, Miklavcic D and Sersa G (1998a) Intrinsic sensitivity of tumor cells to bleomycin as an indicator of tumor response to electrochemotherapy. *Jpn J Cancer Res* **89**: 328–333.

Cemazar M, Sersa G and Miklavcic D (1998b) Electrochemotherapy with cisplatin in treatment of tumor cell resistant to cisplatin. *Anticancer Res* **18**: 4463–4466

Cemazar M, Jarm T, Miklavčič D, Maček-Lebar A, Ihan A, Kopitar NA and Serša G (1998c) Effect of electric-field intesity on electropermeabilization and electrosensitivity of various tumor-cell lines in vitro. *Electro Magnetobiol* **17**: 261–270

Cemazar M, Miklavčič D, Scancar J, Dolzan V, Golouh R and Sersa G (1999) Increased platinum accumulation in SA-1 tumor cells after in vivo electrochemotherapy with cisplatin. *Br J Cancer* **79**: 1386–1391

Clements MK, Jones CB, Cumming M and Daoud SS (1999) Antiangiogenic potential of camptothecin and topotecan. *Cancer Chemother Pharmacol* **44**: 411–416

Denekamp J (1990) Vascular attack as a therapeutic strategy for cancer. *Cancer Metastasis Rev* **9**: 267–282

© 2001 Cancer Research Campaign 

Dirix LY, Libura M, Libura J, Vermeulen PB, Bruijn EA and van Oosterom AT (1997) In vitro toxicity studies with mitomycins and bleomycin on endothelial cells. *Anti-Cancer Drugs* **8**: 859–868

Gilbert RA, Jaroszeski MJ and Heller R (1997) Novel electrode designs for electrochemotherapy. *Biochim Biophys Acta* **1334**: 9–14

Hait WN, Gesmonde JF and Lazo JS (1994) Effect of anti-calmodulin drugs on the growth and sensitivity of C6 rat glioma cells to bleomycin. *Anticancer Res* **14**: 1711–1722

Heller R, Gilbert R and Jaroszeski MJ (2000) Clinical trials of solid tumors using electrochemotherapy. In: *Electrochemotherapy, electrogenetherapy, and transdermal drug delivery. Electrically mediated delivery of molecules to cells*, Jaroszeski MJ, Heller R and Gilbert R (eds) pp 137–156. Humana Press: New Jersey

Kambe M, Arita D, Kikuchi H, Funato T, Tezuka F, Gamo M, Murakawa Y and Kanamaru R (1996) Enhancing the effect of anticancer drugs against the colorectal cancer cell line with electroporation. *Tohoku J Exp Med* **180**: 161–171

Kuriyama S, Matsumoto M, Mitoro A, Tsujinoue H, Toyokawa Y, Nakatani T, Yamazaki M, Okamoto S and Fukui H (1999) Electrochemotherapy against colorectal carcinoma: comparison of in vitro cytotoxicity of 5-fluorouracil, cisplatin and bleomycin. *Int J Oncol* **15**: 89–94

Lazo JS, Braun D, Labaree DC, Schissenbauer JC, Meandzija B, Newman RA and Kennedy KA (1989) Characteristics of bleomycin-resistant phenotypes of human cell sublines and circumvention of bleomycin resistance by liblomycin. *Cancer Res* **49**: 185–190

Miklavcic D, Beravs K, Semrov D, Cemazar M, Demsar F and Sersa G (1998) The importance of electric field distribution for effective *in vivo* electroporation of tissues *Biophys J* **74**: 2152–2158

Mir LM and Orlowski S (2000) The basis of electrochemotherapy. In: *Electrochemotherapy, electrogenetherapy, and transdermal drug delivery. Electrically mediated delivery of molecules to cells*, Jaroszeski MJ, Heller R and Gilbert R (eds) pp 99–117. Humana Press: New Jersey

Mir LM, Roth C, Orlowski S, Quintin-Colonna F, Fradelizi D, Belehradek JJr and Kourilsky P (1995) Systemic antitumor effects of electrochemotherapy combined with histoincompatible cells secreting interleukin-2. *J Immunother* **17**: 30–38

Orlowski S, Belehradek J Jr, Paoletti C and Mir LM (1998) Transient electropermeabilization of cells in culture. Increase of the cytotoxicity of anticancer drugs. *Biochem Pharmacol* **37**: 4727–4733

Rols MP and Teissie J (1998) Flow cytometry quantification of electropermeabilization. In: *Methods in molecular biology, Vol 91: Flow cytometry protocols*, Jaroszeski MJ and Heller R (eds) pp 141–147. Humana Press: New Jersey

Sersa G (2000) Electrochemotherapy: animal work review. In: *Electrochemotherapy, electrogenetherapy, and transdermal drug delivery. Electrically mediated delivery of molecules to cells*, Jaroszeski MJ, Heller R and Gilbert R (eds) pp 119–136. Humana Press: New Jersey

Sersa G, Cemazar M and Miklavcic D (1995) Antitumor effectiveness of electrochemotherapy with cis-diamminedichloroplatinum (II) in mice. *Cancer Res* **55**: 3450–3455

Sersa G, Miklavcic D, Cemazar M, Belehradek J Jr, Jarm T and Mir LM (1997a) Electrochemotherapy with CDDP on LPB sarcoma: comparison of the anti-tumor effectiveness in immunicompetent and immunodeficient mice. *Bioelectroch Bioener* **43**: 297–283

Sersa G, Cemazar M, Menart V, Gaberc-Porekar V and Miklavcic D (1997b) Antitumor effectiveness of electrochemotherapy is increased by TNF-$\alpha$ on SA-1 tumors in mice. *Cancer Letters* **116**: 85–92

Sersa G, Cemazar M, Parkins CS and Chaplin DJ (1999a) Tumour blood flow changes induced by application of electric pulses, *Eur J Cancer* **35**: 672–677

Sersa G, Cemazar M, Miklavcic D and Chaplin DJ (1999b) Tumor blood flow modifying effect of electrochemotherapy with bleomycin. *Anticancer Res* **19(5B)**: 4017–4022

 © 2001 Cancer Research Campaign
